# Enhancing Volume Precision in Breast Reconstruction: A BMI-Based Model for Predicting Flap Weight in Profunda Artery Perforator Flaps

**DOI:** 10.3390/life15040667

**Published:** 2025-04-17

**Authors:** Charalampos Varnava, Shaghayegh Gorji, Sascha Wellenbrock, Tobias Hirsch, Maximilian Kückelhaus, Philipp Wiebringhaus

**Affiliations:** 1 Department of Plastic Surgery, University Hospital Muenster, 48149 Muenster, Germany; 2 Department of Plastic, Reconstructive and Aesthetic Surgery, Hand Surgery, Fachklinik Hornheide, 48157 Muenster, Germany; 3 Institute of Musculoskeletal Medicine, University Hospital Muenster, 48149 Muenster, Germany

**Keywords:** volumetry, PAP flap, breast reconstruction, quality of life, breast cancer

## Abstract

**Background:** In recent years, the profunda artery perforator (PAP) flap has gained recognition as a viable technique in autologous breast reconstruction, demonstrating low donor site morbidity and favorable reconstructive outcomes. This study aims to highlight the recent refinements using the PAP flap for breast reconstruction focusing on preoperative volume prediction. **Methods:** A retrospective study was conducted to evaluate the outcomes of breast reconstruction surgeries using the PAP flap at our institution between May 2018 and December 2022. A total of 114 PAP flaps performed in 96 patients were included. Statistical analysis was performed to analyze flap volume in relation to body mass index (BMI). Surgical details, such as donor site-related complications and patient characteristics, were also collected. **Results:** BMI was identified as a statistically significant predictor (*p* < 0.001) for flap weight in the resulting model. The average BMI observed was 23.0, while the mean flap weight was 304.2 g. The predictive model for flap weight was determined as follows: flap weight (g) = (19.252 × BMI) − 143.572. This model underscores the relationship between BMI and flap weight, quantifying the influence of BMI on flap weight prediction. **Conclusions:** Our study indicates that it is feasible to harvest an adequate volume for breast reconstruction even in patients with a low BMI. To facilitate preoperative prediction of PAP flap volume, we developed an algorithm designed to enhance preoperative planning and estimate the need for supplementary procedures to achieve the desired volume.

## 1. Introduction

In 2020, the World Health Organization (WHO) reported that 2.3 million women worldwide were diagnosed with breast cancer, establishing it as the most common malignant neoplasm among women globally [1]. Age-standardized breast cancer mortality in high-income countries dropped by 40% between the 1980s and 2020. Consequently, there has been a notable rise in the frequency of breast reconstruction surgeries, driven by advancements in surgical techniques, increased awareness of post-mastectomy options, and the growing emphasis on improving patients’ quality of life and psychosocial well-being following breast cancer treatment. Among the primary reconstructive approaches—autologous and implant-based reconstruction—autologous reconstruction often provides numerous advantages over implant-based surgery for many patients. Heterologous breast reconstruction is still the most common type of reconstructive procedure if nipple and skin sparing mastectomies are performed. Recent studies show significant improvement in the latest generations of implants [2]. Nevertheless, autologous reconstruction is particularly advantageous in producing a more natural outcome and eliminates the need for heterologous materials like implants or acellular dermal matrixes (ADM), thereby reducing the risk of complications such as capsular contracture, implant rupture, or displacement [3]. Additionally, there have been significant advances in microsurgery in recent years, leading to refinements of surgical techniques with decreased operative times, reduced morbidity, and improved patient-reported outcomes [4]. Currently, the deep inferior epigastric perforator (DIEP) flap is the flap of choice in patients opting to undergo autologous breast reconstruction [5]. The DIEP flap has emerged as an excellent option for direct-to-autologous tissue reconstruction following prophylactic mastectomies, particularly in patients with larger breast volumes, where implant-based reconstructions are limited. For individuals with larger breast sizes, the DIEP flap provides sufficient volume to achieve esthetically pleasing and symmetrical outcomes, making it a preferred choice in reconstructive surgery. Its ability to combine robust vascularity with minimal functional compromise further underscores its suitability in this patient population [6]. However, abdominal-based flaps may be contraindicated or insufficient in certain cases, such as in patients with a history of abdominal surgeries or those with low body fat, where minimal abdominal soft tissue limits the available donor material. The profunda artery perforator (PAP) flap presents a strong alternative as a thigh-based flap due to its reliable blood supply and muscle-sparing nature. Studies report similar patient-related and flap-related outcomes for the PAP flap compared to abdominal-based flaps [7,8]. Other autologous alternatives, such as the lumbar artery perforator (LAP) flap, as secondary options indicate lower success rates [9]. Several authors have noted benefits such as reduced operative times and expedited postoperative ambulation [10].

One of the main disadvantages of the PAP flap is its relatively limited volume and skin for larger reconstructions when used as a single flap. Common solutions to address limited volume include utilizing stacked flaps harvested from bilateral thigh donor sites or performing subsequent lipofilling procedures. The objective of this study was to develop an algorithm for preoperative prediction of PAP flap volume, with the goal of optimizing preoperative planning and assessing the potential need for additional procedures, such as lipotransfer, to achieve sufficient volume.

## 2. Materials and Methods

### 2.1. Study Population

This study was a retrospective analysis conducted to assess the outcomes of breast reconstruction surgeries utilizing PAP flaps performed at our institution from May 2018 to December 2022. The study included a total of 114 PAP flap reconstructions, comprising 75 unilateral cases, 16 bilateral cases, and one stacked PAP case. Inclusion criteria encompassed patients who underwent PAP flap procedures for breast reconstruction and had complete preoperative and postoperative records available for analysis. Exclusion criteria included any patients who received combined flap techniques other than the specified stacked PAP or had incomplete follow-up data. Ethical approval was obtained from our institution’s review board prior to study initiation.

### 2.2. Operating Technique

**Figure 1:**
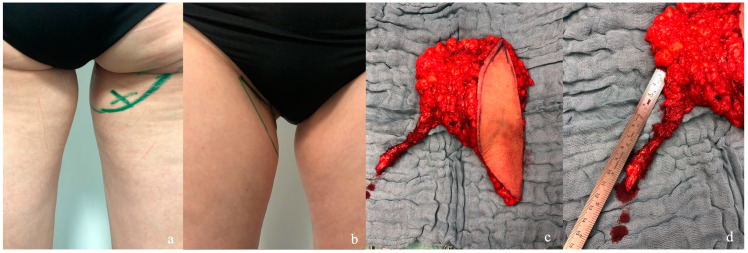
(**a**) Preoperative marking after location of the dominant perforator by duplex ultrasound, dorsal view. (**b**) Ventral view. (**c**) Harvested flap including a small paddle with perforator located outside. (**d**) Average pedicle length in our cohort was 10.5 cm ± 1.4 cm. Image is reproduced from Varnava C. et al. [9].

Flap harvest was performed in supine position. Preoperatively the dominant perforator was located by duplex ultrasound for location, flow, and size determination (Figure 1). A pinch test was performed to determine the amount of skin resection. Cranial incision was made in the gluteal fold, whereas the distal incision was determined by the location of the main perforator.

During preparation of the flap, attention was given to maximize the volume of the flap by harvesting wide distal fat from the flap’s distal incision.

**Figure 2:**
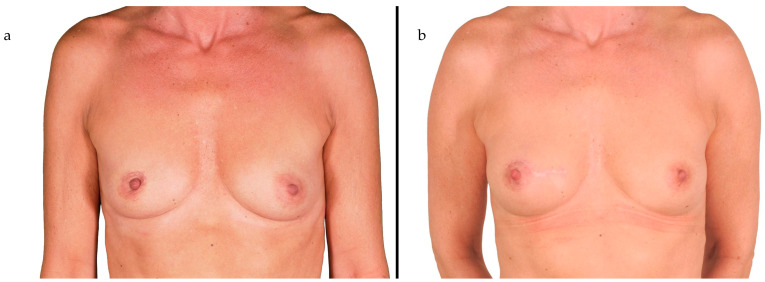
(**a**) Preoperative and (**b**) postoperative image of a 54-year-old patient with multifocal ductal carcinoma in situ of the right breast who underwent a nipple-sparing mastectomy and immediate autologous reconstruction using a PAP flap at our institution. Image is reproduced from Varnava C. et al. [9].

By using this technique, a maximum amount of fat could be included in the flap design. Due to its robust and consistent perforasome, the profunda artery perforator can often be located outside the skin island for a better skin closure when little to no skin for breast reconstruction is needed, e.g., after nipple-sparing mastectomy (NSM) (Figure 2) [10].

The flap inset into the breast was achieved by anastomosing the PAP flap to the internal mammary artery and its accompanying vein. In certain cases, both veins of the flap were utilized, and venous anastomoses were performed using a microvascular anastomotic coupler (Synovis Micro Companies Alliance, Inc., Birmingham, AL, USA) to ensure optimal vascular outflow [11]. In case of the stacked PAP flap, inset was obtained by using the internal mammary artery and vein in an ante- and retrograde way.

### 2.3. Data Collection and Processing

Patient demographic and clinical data were systematically gathered from electronic health records and recorded in a database. The collected parameters included the patients’ age in years at the time of surgery and sex, which was recorded for completeness; however, all identified patients were female. The BMI was calculated based on preoperative height and weight data (kg/m^2^). Additionally, the ASA score was recorded to assess general health status and perioperative risk.

Comorbidities such as diabetes, hypertension, and other relevant medical conditions were documented. Intraoperative metrics included flap ischemia time, defined as the duration of blood flow interruption to the flap tissue in minutes, as well as the total operative time from the initial incision to closure.

Postoperative outcomes were recorded, focusing on flap-related complications, including partial or total flap loss, as well as donor site complications such as delayed wound healing or infections. Furthermore, any revision procedures were noted if additional interventions or corrective surgeries were required in relation to flap outcomes.

All data were initially entered and organized in Microsoft Excel (Redmond, WA, USA), which facilitated preliminary sorting and verification.

### 2.4. Statistical Analysis

Statistical analysis was carried out using IBM SPSS Statistics software (version 26, Armonk, NY, USA). The primary analysis aimed to explore the relationship between BMI and flap weight, with secondary analyses conducted to examine other potential predictors of flap outcomes.

A simple linear regression analysis was conducted to evaluate whether BMI could predict flap weight as the dependent variable. A regression model was fitted with BMI as the independent variable and flap weight as the dependent variable. The overall significance of the model was assessed using an F-test, while a *t*-test was performed on the BMI coefficient to determine the strength and significance of its predictive value. The accuracy of predictions was evaluated using the mean of absolute residuals.

To further investigate whether model accuracy could be improved, a multiple linear regression analysis was performed by including age as an additional predictor alongside BMI. However, age did not significantly enhance the model, as it lacked statistical significance. Consequently, age was excluded from the final model. For all statistical tests, a significance threshold of *p* < 0.05 was applied to determine statistical relevance.

Results were interpreted in the context of clinical implications for breast reconstruction outcomes and the impact of BMI on flap viability and complications.

## 3. Results

A total of 114 PAP flaps were performed in 96 patients undergoing autologous breast reconstruction with PAP flap. The mean age at surgery was 46.5 ± 11.9 years and mean body mass index was 23.0 ± 3.6 kg/m^2^. A total of 76 patients (79.2%) had mild systemic diseases (ASA II), while the rest were healthy (ASA I). We performed 78 unilateral and 34 bilateral PAP flaps. One patient received a stacked PAP breast reconstruction (Table 1).

The mean flap weight was 304.2 ± 100.2 g. Mean ischemia time was 57.7 ± 33.7 min including both bilateral and unilateral PAP flaps. The flap survival rate was 97.4%. The most common donor site complication was delayed wound healing, occurring in 37.7% of cases, followed by seroma (7.9%) and infection (7.0%). The mean inpatient stay was 6.7 ± 2.2 days. Twenty-eight breasts (24.8%) required subsequent lipotransfer (Table 2).

A simple linear regression with the BMI as predictor significantly predicted flap weight as the dependent measure, F (1.96) = 98.637, *p* < 0.001, explaining 50.7% of flap weight variance (r^2^ = 0.507). BMI was shown to be a significant coefficient (t(96) = 9932; *p* < 0.001).

The resulting model was determined as follows:

flap weight (g) = (BMI × 19.252) − 143,572

**Figure 3:**
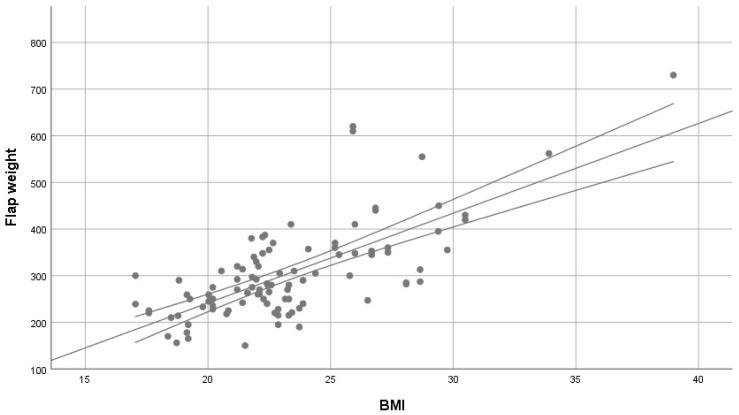
Scatter plot for BMI and flap weight with regression line. Top and bottom lines indicate individual 95% confidence intervals.

The mean of the absolute residuals of this model was 52.0859 g, 95% CI [42.65 g, 61.52 g] (Figure 3). A model with BMI and age as additional predictors did not demonstrate significance for age.

## 4. Discussion

The PAP flap has become a standard alternative in autologous breast reconstruction for patients for whom abdominal-based free flaps are insufficient. With proper preoperative planning and surgical techniques, the PAP flap is a well-suited option with high reconstructive success rates and excellent esthetic outcomes, as well as comparable donor site morbidity [12,13].

Quantitative evaluations of breast volume loss following autologous breast reconstruction are relatively scarce in the literature. Several studies have employed computer tomography (CT) imaging to assess anatomical structures, measure volumetric parameters, and predict the gradual reduction in flap volume over time [14,15]. Although CT-based volumetric analysis offers accurate and detailed measurements, its routine clinical application is constrained by significant drawbacks, including radiation exposure and high associated costs. Consequently, non-invasive and lower-risk alternatives are often preferred for breast volume assessment. Magnetic resonance imaging (MRI)-based volumetry has also been utilized to provide more detailed information in both autologous and implant-based reconstructions [16]. However, the high costs and limited accessibility of MRI technology present challenges for widespread adoption in clinical practice. Moreover, standardized volumetric algorithms for outcome evaluation remain largely undeveloped [17]. While three-dimensional (3D) scanning technology delivers fairly accurate results, its use in clinical settings is limited due to its high cost and lack of portability. For many healthcare facilities, particularly smaller clinics, the substantial upfront investment and maintenance requirements render this technology impractical. Additionally, the bulky design of most 3D scanners makes them unsuitable for routine use in fast-paced or dynamic clinical environments. The absence of user-friendly, portable options further restricts their application, especially in outpatient or rural settings. Consequently, traditional diagnostic tools continue to be the preferred choice, despite their inherent limitations [18].

In our experience, around one third of the patients who underwent autologous breast reconstruction using the PAP flap require supplemental refinements to add volume with lipotransfer (Table 1). Autologous fat grafting has become a crucial tool within breast surgery as it is simple to obtain with minimal surgical trauma and a natural esthetic outcome. Laporta et al. reported that satisfaction was higher among patients who underwent autologous reconstruction with additional lipotransfer compared to patients without fat grafting [19]. However, the long-term outcomes vary due to differences in absorption rate with reports ranging from 20 to 90% and therefore largely unpredictable results [20]. Another notable drawback is the lack of conclusive scientific evidence regarding the potential of lipotransfer to induce breast cancer. Although clinical studies have not demonstrated a direct link between lipotransfer and breast cancer development, concerns persist due to findings from in vitro studies. These studies suggest that adipocyte-derived stem cells may promote the proliferation or activation of certain cancer cell populations under specific conditions [21,22]. This uncertainty raises questions about the long-term safety of lipotransfer, particularly in patients with a history of breast cancer or those considered at high risk. As a result, some breast surgeons do not perform consecutive lipotransfers after breast cancer. Therefore, the need for more precise preoperative planning, including accurate volume prediction, may enhance the reliability of estimating volumetric outcomes and the potential need for subsequent surgeries, while also enabling clearer communication of expectations to patients. In addition, the preoperative estimation could contemplate the possible stacking of flaps or the use of hybrid solutions combining implant-based and autologous reconstructions [23,24].

Using the BMI to predict flap volume, we established a readily available, non-invasive tool to achieve relatively high precision. Furthermore, our non-invasive method provides a more individualized and precise estimation, enhancing preoperative planning and patient outcomes. By utilizing BMI, our algorithm simplifies the process and reduces the need for complex and costly equipment.

In summary, recent advancements in microsurgical breast reconstruction have led to a shift in the definition of success from sole flap survival to refinements in esthetics at both recipient and donor sites. Aiming for optimally tailored donor-site selection and flap design that adapts to the patients’ individual body shapes has led to more individualized configurations of stacked, bipedicled, and conjoined thigh-based flaps with the introduction of the L-PAP as one recent example [25,26,27,28,29]. Incorporation of these concepts into flap design for breast reconstruction enables an efficient and individualized use of available tissues to address reconstructive needs.

Our experience supports the growing body of evidence on the safety and efficacy of the PAP flap as a robust alternative to the DIEP flap for autologous breast reconstruction. Moreover, the proposed algorithm may enhance the accuracy of volumetric outcome estimations based solely on patients’ BMI. The presented algorithm is routinely employed in patients undergoing autologous breast reconstruction with PAP flaps in our institution. It has proven to be an invaluable tool for preoperative planning, facilitating precise surgical preparation and enhancing communication to address patients’ specific needs and expectations effectively.

The limitations of the study include our sample size, which may not be generalizable to diverse populations, and the single-center data collection, which could introduce bias specific to our institution’s practices. The model primarily uses BMI as a predictor for flap weight, leaving out other potentially relevant factors such as body composition and comorbid conditions. While our model captures a significant portion (51.1%) of the variability in flap weight, a portion remains unexplained, suggesting that additional variables such as body morphology may also be important.

The mean absolute residuals reveal significant variability in the predictions, and the retrospective design of the study introduces inherent biases. Furthermore, the model has not been externally validated, limiting its generalizability.

## 5. Conclusions

Advancements in microsurgical techniques and preoperative planning tools continue to refine autologous breast reconstruction, shifting the focus to achieving optimal esthetic and functional outcomes. Our study contributes to this evolving field by providing a practical method for optimizing PAP flap utilization, further enhancing reconstructive outcomes and patient satisfaction. Future research should address the limitations in this study by incorporating larger, more diverse samples, considering additional predictive variables, and employing multi-center and prospective study designs.

## Figures and Tables

**Table 1:** Patient characteristics and comorbidities.

Patient Characteristics	Value
Age, yr	
Mean ± SD	46.4 ± 11.9
Range	17–77
Sex (n)	
Male	0
Female	96
BMI (body mass index)	
Mean ± SD (kg/m^2^)	23.0 ± 3.6
Range	17–39
**Comorbidities (%)**	
ASA class	
1	20.8
2	79.2
3	0
Hypertension	7.3
Diabetes mellitus	0
Smoker (active)	9.4
**Type of flap (n)**	
Unilateral PAP	78
Bilateral PAP	34
Stacked PAP	2

**Table 2:** Surgical details, flap survival, and complications.

Ischemia Time, Min	Value
Mean	57.7 ± 33.7
Range	19–223
**Flap Weight, g**	304.2 ± 100.2
**Pedicle Length, cm**	
Mean	10.4 ± 1.4
Range	7–14
**Flap Complications (%)**	
Anastomosis revision rate	2.6
Flap loss	2.6
Survival rate	97.4
**Donor Site Complications (%)**	
Delayed wound healing	37.7
Seroma	7.9
Infection	7.0
Wound dehiscence	5.3
**Lipotransfer (%)**	24.8
**Inpatient Stay Length (d)**	
Mean	6.7 ± 2.2
Range	3–17

## Data Availability

Data available on request.
